# Immune Checkpoint Inhibitors for Lung Cancer Treatment: A Review

**DOI:** 10.3390/jcm9051362

**Published:** 2020-05-06

**Authors:** Keisuke Onoi, Yusuke Chihara, Junji Uchino, Takayuki Shimamoto, Yoshie Morimoto, Masahiro Iwasaku, Yoshiko Kaneko, Tadaaki Yamada, Koichi Takayama

**Affiliations:** Department of Pulmonary Medicine, Graduate School of Medical Science, Kyoto Prefectural University of Medicine, Kyoto 602-8566, Japan; onoi@koto.kpu-m.ac.jp (K.O.); c1981311@koto.kpu-m.ac.jp (Y.C.); m04035ts@koto.kpu-m.ac.jp (T.S.); yoshie-m@koto.kpu-m.ac.jp (Y.M.); miwasaku@koto.kpu-m.ac.jp (M.I.); kaneko-y@koto.kpu-m.ac.jp (Y.K.); tayamada@koto.kpu-m.ac.jp (T.Y.); takayama@koto.kpu-m.ac.jp (K.T.)

**Keywords:** immune checkpoint inhibitors, non-small-cell lung cancer, PD-1, biomarker

## Abstract

The treatment of lung cancer has changed drastically in recent years owing to the advent of immune checkpoint inhibitors (ICIs). A 1992 study reported that programmed cell death-1 (PD-1), an immune checkpoint molecule, is upregulated during the induction of T cell death. Since then, various immunoregulatory mechanisms involving PD-1 have been clarified, and the successful use of PD-1 blockers in anticancer therapy eventually led to the development of the current generation of ICIs. Nivolumab was the first ICI approved for treating lung cancer in 2014. Since then, various ICIs such as pembrolizumab, atezolizumab, and durvalumab have been successively introduced into clinical medicine and have shown remarkable efficacy. The introduction of ICIs constituted a major advancement in lung cancer treatment, but disease prognosis continues to remain low. Therefore, new molecular-targeted therapies coupled with existing anticancer drugs and radiotherapy have recently been explored. This review encompasses the current status, challenges, and future perspectives of ICI treatment in lung cancer.

## 1. Introduction

Among all malignancies, lung cancer showed the highest reported incidence and mortality in 2018 [1]. The prognosis of advanced and recurrent lung cancer is poor, and standard treatments with cytotoxic anticancer drugs have limited therapeutic effects. Recently, with the development of molecularly targeted drugs based on the results of genetic testing and immunotherapies for cancer, treatments for non-small cell lung cancer have undergone remarkable development. Molecularly targeted drugs for cancer can differentiate between cancer cells and normal cells at the genome and molecule levels and act by specifically suppressing the molecules required for cancer growth and metastasis. Cytotoxic drugs are different as they have defined molecular targets from the stage of drug discovery and therapy design, and their targets are often biomarkers, especially those predicted for treatment. In non-small cell lung cancer (NSCLC), driver oncogene mutations, which confer advantages to the growth and viability of cancer cells, are the mainstay of biomarkers. EGFR gene mutations, ALK gene translocations, ROS1 gene translocations, and BRAF gene mutations have been used, and tyrosine kinase inhibitors targeting these aberrations have elicited high response rates.

Since around 1970, immunotherapy has been initiated for lung cancer with nonspecific treatments such as OK-432, and has progressed to specific immunotherapies such as peptide vaccine therapy. However, no treatment has shown apparent efficacy beyond the standard of care with cytotoxic anticancer drugs. In recent years, as the detailed mechanism of tumor immunotherapy is understood, and anti PD-1 antibodies, one of the immune checkpoint inhibitors, have shown good results in clinical trials with increasing insurance support, the treatment of lung cancer has entered a new era. Several trials in advanced NSCLC have reported improved survival with anti-PD-1/PD-L1 antibodies treatment, both when used alone and in combination with chemotherapy (Table 1). This article thus elaborates on the immune checkpoint inhibitors used for treating lung cancer.

## 2. Mechanism of Action of Immune Checkpoint Inhibitors (ICIs)

Although cancer cells are formed daily, almost all of them are properly eliminated through the host immune response. Immune responses to cancer cells are called cancer-immunity cycles and comprise seven phases: (1) release of cancer antigens by the death of cancer cells, (2) presentation of cancer antigens to T cells by antigen-presenting cells such as dendritic cells, (3) T cell activation (priming phase), (4) T cell migration, (5) T cell infiltration, (6) cancer cell recognition, and (7) attack and elimination of cancer cells (effector phase) [2]. However, cancer cells with low immunogenicity, which do not present cancer antigens, may evade this autoimmune response and survive for a longer duration (equilibrium phase) [2,3]. Further, immunosuppressive mechanisms activated upon the accumulation of mutations in cancer cells, the induction of regulatory T cells (Tregs) and immunosuppressive cells including myeloid-derived suppressor cells (MDSCs), and the expression of immune checkpoint molecules such as PD-L1 result in uncontrolled tumor growth (escape phase) [2,3]. Thus, certain cancers are detected only after the cancer cells approach the escape phase and undergo uncontrolled proliferation, having already established a system preventing them from being eliminated through the autoimmune response.

**Figure 1:**
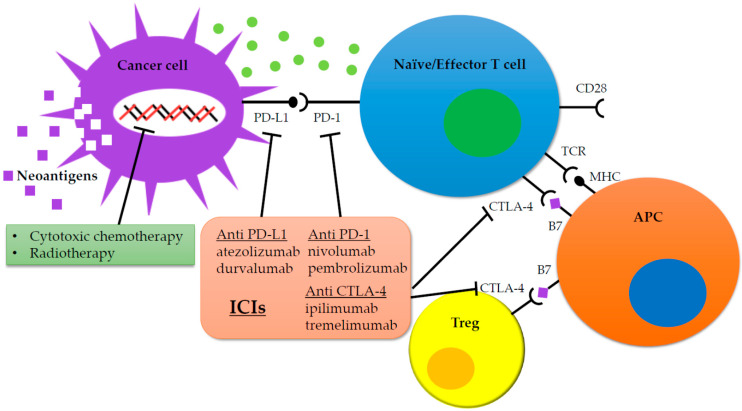
Immune checkpoint inhibitors in cancer treatment. Notes: Inability to activate T cells in the tumor microenvironment through the suppressive effect of Tregs or through immune checkpoints allows cancer cells to escape immune attack, survive, and grow. B7 ligands expressed on antigen-presenting cells bind to TCR and induce T cell amplification and immune response. Alternatively, binding of B7 ligands to CTLA-4 expressed on T cells suppresses their activity. CTLA-4 also enhances the activity of Tregs leading to immunosuppressive activity. PD-1 is expressed on activated T cells. PD-1 binds to its PD-L1 leading to the anergy of T cells, further promoting inhibitory signals. Pharmacological inhibition of immune checkpoints with monoclonal antibodies restores T cell antitumor activity and relieves immunosuppression. Abbreviations: CTLA-4: cytotoxic T-lymphocyte antigen 4; MHC: major histocompatibility complex; PD-1: programmed cell death-1; PD-L1: programmed cell death-1 ligand; TCR: T cell receptor; Tregs: regulatory T cells; APC: antigen presenting cell.

ICIs are drugs that block the immunosuppressive mechanisms of cancer cells (Figure 1). ICIs exert their antitumor effects by harnessing host autoimmune functions, as opposed to cytocidal anticancer drugs, which inhibit the cell cycle, and agents that directly attack cancer cells, such as molecularly targeted drugs that specifically bind to gene mutation sites and suppress proliferative signals. Currently, anti-PD-1/PD-L1 antibodies are clinically used to treat lung cancer and various other cancers. In lung cancer, PD-L1 expression is used as one of the biomarkers to distinguish the treatment indication cases. Microsatellite instability has also been used as a potential anti-PD-1/PD-L1 antibodies treatment biomarker in gastric cancer, mainly as a second-line treatment after standard treatment, in triple-negative breast cancer, and as a biomarker candidate in colorectal cancer. In 2011, monotherapy with ipilimumab, an anticytotoxic T-lymphocyte antigen 4 (CTLA-4) antibody, was approved by the food and drug administration (FDA) for advanced-stage malignant melanoma, and in 2015, the combination of nivolumab and ipilimumab was approved by the FDA for use in clinical practice. Studies comparing ipilimumab+nivolumab with sunitinib alone in renal cell carcinoma and the combination of ipilimumab+nivolumab in non-small cell lung cancer have shown favorable results [4,5]. Regarding the significance of ipilimumab in combination therapy, future results are awaited as to whether two-drug combinations of immune checkpoint inhibitors (ipilimumab and nivolumab combination therapy) can contribute to higher survival rates than either immune checkpoint inhibitors alone or immune checkpoint inhibitors in combination with chemotherapy. Since numerous aspects of the mechanism of action of ICIs in vivo are unclear, this review discusses the generally considered mechanisms.

Anti-PD-1/PD-L1 antibodies act in the effector phase of the cancer-immunity cycle. In the effector phase, effector T cells attack cancer cells. However, binding of PD-L1 expressed on the cancer cell surface to PD-1 expressed on the surface of effector T cells suppresses the attack by effector T cells on cancer cells. Anti-PD-1/PD-L1 antibodies pharmacologically prevent the PD-1/PD-L1 interaction, thus facilitating the attack by T cells. Furthermore, these antibodies are thought to inhibit the immune response in the priming phase of the cancer-immunity cycle [6].

In contrast, anti-CTLA-4 antibodies act during antigen presentation in the priming phase, wherein dendritic cells present antigens to and activate T cells. T-cell activation requires both T-cell receptors (TCRs) and the MHCI-cancer antigen complex on the dendritic cells (principal stimulation), accompanied by the interaction between B7 (CD80/86) and CD28 on dendritic and T cells, respectively (costimulation) [7]. CTLA-4, like CD28, is expressed on the T cell surface and binds B7 with a stronger affinity than that of CD28. Thus, when CTLA-4 is upregulated, it remains bound to B7 and the costimulatory signal is not transmitted, resulting in the suppression of T cell activation [8]. Anti-CTLA-4 antibodies inhibit the binding of CTLA-4 and B7, resulting in enhanced binding of CD28 and B7, which stimulates T-cell activation and exerts antitumor effects (Figure 1) [9]. Furthermore, CTLA-4 is present on Treg surfaces, induced by cancer cells, and inhibits T-cell activation by binding to B7 on dendritic cells [10]. Thus, anti CTLA-4 antibodies are also thought to exert antitumor effects by facilitating the binding of Tregs to CTLA-4 and directly eliminating Tregs.

## 3. Changes in Treatment of Lung Cancer without Driver-Oncogene Mutations

Second-line therapy following platinum-based chemotherapy has long been cytotoxic therapies such a docetaxel (DTX).

In 2014, nivolumab, the world’s first ICI targeting PD-1, emerged as a novel therapeutic agent for malignant melanoma. In 2015, a phase-III comparative study of DTX and nivolumab as secondary treatments for squamous and non-squamous lung cancers was conducted in the CheckMate017 (NCT01642004) and CheckMate057 (NCT01673867) studies, respectively; both studies reported that nivolumab significantly prolonged overall survival (OS) compared to DTX (CheckMate017: 6.0 mo vs. 9.2 mo, Hazard Ratio (HR) 0.59; CheckMate057: 9.4 mo vs. 12.2 mo, HR 0.73) [11,12]. Considering these findings, the indication of nivolumab was also expanded to the second-line treatment of NSCLC, and ICIs were approved for the first time for lung cancer treatment.

In 2016, another anti-PD-1 antibody, pembrolizumab, has been reported, and a phase-III comparative study of DTX and pembrolizumab as second-line therapy in NSCLC with PD-L1 ≥ 1% reported that pembrolizumab significantly prolonged patient survival compared to DTX (8.5 mo vs. 10.4 mo (pembro 2 mg/kg) /12.7 mo (pembro 10 mg/kg) [13]. Furthermore, the OAK trial (NCT02008227) compared the second-line NSCLC anti PD-L1 antibodies atezolizumab and DTX, and showed that atezolizumab prolonged survival significantly (9.6 mo vs. 13.8 mo, HR 0.73) [14]. Based on these results, pembrolizumab and atezolizumab, in addition to nivolumab, were introduced as the second-line treatment for NSCLC.

Subsequent to being established as a standard-of-care treatment for second-line therapy, in the KEYNOTE-024 study (NCT02142738) (2016), pembrolizumab significantly prolonged the overall survival (OS) of patients (10.3 mo vs. 6.0 mo, HR 0.60) [15] upon platinum-based chemotherapy as first-line therapy in a PD-L1 ≥ 50% NSCLC without driver mutations, and was approved for the first time as first-line treatment for NSCLC. In 2018, KEYNOTE-189 trial (NCT02578680) and KEYNOTE-407 trials (NCT02775435) assessed the efficacy of the combination of platinum-based chemotherapy and ICIs and approved this combination therapy as first-line treatment of lung cancer, as it significantly prolonged the OS compared to platinum-based chemotherapy alone (KEYNOTE-189 not reached (NR) vs. 11.3 mo, HR 0.49; KEYNOTE-407 15.9 mo vs. 11.3 mo, HR 0.64) [16,17] by combining pembrolizumab with platinum-based chemotherapy for non-squamous-cell lung cancer and squamous cell lung cancer, respectively. In the same year, maintenance therapy with chemoradiotherapy (CRT) followed by durvalumab drastically improved the progression-free survival (PFS) in comparison with CRT alone in unresectable stage III NSCLC in the PACIFIC study among patients with locally advanced lung cancer (16.8 mo vs. 5.6 mo, HR 0.52) [18], and thus, ICIs contributed to advancements in the standard-of-care treatment for locally advanced NSCLC for the first time in 20 y.

In 2019, the IMpower133 trial (NCT02763579) reported that the combination of atezolizumab with platinum-based chemotherapy, as first-line treatment of small-cell lung cancer (SCLC), prolonged both the PFS and OS (PFS 4.3 mo vs. 5.2 mo, HR 0.77; OS 10.3 mo vs. 12.3 mo, HR 0.70) [19]. ICIs are expected to be used to treat SCLC.

Thus, since 2016, ICIs have been widely used therapeutics in different settings from first-line to second-line and onwards, for locally advanced to advanced-stage NSCLC and SCLC and lung cancer.

## 4. Immune Combination Therapy

### 4.1. Combination with Chemotherapy

To enhance the efficacy of immunotherapy, the combination of platinum-based chemotherapy and ICIs has already been validated and introduced into actual clinical practice (Figure 1). As one of them, a comparative study of CDDP or CBDCA+Pemetrexed (PEM) plus pembrolizumab vs. CDDP or CBDCA+PEM was conducted in the KEYNOTE-189 trial, and the PFS was significantly higher in the ICI-combined group than in the chemotherapy group in PFS (8.8 mo vs. 4.9 mo, HR 0.52) and OS (NR vs. 11.3 mo, HR 0.49) [16], and this regimen was approved as first-line treatment for advanced-stage NSCLC. This study included a platinum-combination therapy, which has not been directly compared with ICI alone, and it thus remains controversial whether pembrolizumab alone or ICI plus chemotherapy is beneficial for patients with high PD-L1 expression levels. Upon ICI monotherapy, cases presenting with cancer progression at an early stage pose a problem, whereas during combination therapy, it is advantageous to reduce progressive disease at early stages in combination treatment with cytotoxic anticancer drugs.

The therapeutic efficacy of atezolizumab as first-line therapy for NSCLC was reported in an IMpower150 trial (NCT02366143) in combination with CBDCA+paclitaxel (PTX)+bevacizumab (BEV) [19]. In this study, subgroup analyses confirmed the efficacy of ICIs among patients with hepatic metastases and driver mutations, which were previously poor responders to ICIs, suggesting the potential effects of the concomitant use of angiogenesis inhibitors. Combination therapy with angiogenesis inhibitors is expected in this regimen in cases with complications including cerebral edema and pleural effusion caused by brain metastasis.

Subsequently, the efficacy of CBDCA+nabPTX in combination with pembrolizumab was also reported in squamous cell carcinoma in a KEYNOTE-407 trial (NCT02775435) [17]; furthermore, the combination treatment with CBDCA+nabPTX and atezolizumab [20] yielded better outcomes than chemotherapy during first-line treatment of squamous cell lung cancer in the IMpower130 trial (NCT02367781). Clinical trials are currently underway for numerous combinations of ICIs and chemotherapy, including the IMpower132 trial (NCT02657434; CBDCA+PEM+atezolizumab versus CBDCA+PEM in non-squamous lung cancer), TORG1630 trial (UMIN000021813; DTX+nivolumab versus DTX alone in NSCLC), KEYNOTE-604 trial (NCT03066778; pembrolizumab+etoposide+carboplatin/cisplatin (EP) versus placebo+EP in SCLC), and CASPIAN trial (NCT03043872; durvalumab+tremelimumab+EP versus durvalumab+EP in SCLC).

Based on these results, various combinations of ICI plus platinum-based chemotherapy, ICI alone, and chemotherapy alone seemed appropriate as first-line treatment of advanced-stage NSCLC at present. It is thus important to examine the optimum treatment for each case on the basis of the factors including the performance status, PD-L1 expression rate, presence of driver gene mutations, and medical history.

### 4.2. Combination of ICIs

Combination therapy with different ICIs is currently being assessed, and the promising regimens include nivolmab+ipilimumab and durvalumab+tremelimumab. These studies have attempted to enhance the antitumor efficacy of immune cells by combining the inhibitors of PD-1/PD-L1 in the effector phase, using inhibitors of CTLA-4 in the priming phase.

In the CheckMate227 trial, a controlled trial involving combination therapy with nivolumab+ipilimumab and chemotherapy was conducted in 2019, and nivolumab+ipilimumab resulted in a significantly better OS among patients with PD-L1 ≥ 1% (17.1 mo vs. 14.9 mo, HR 0.79) [21]. Accordingly, combination therapy with an anti PD-1 antibody and anti CTLA-4 antibody can be clinically introduced for the first time. The POSEIDON trial (NCT03164616; durvalumab+tremelimumab+platinum-based chemotherapy) examining is also ongoing.

Alternatively, higher rates of immune-related adverse events (irAE) have been reported upon ICIs combination therapy. In studies including other carcinomas, anti CTLA-4 antibodies are reportedly associated with a higher incidence of grade-III or higher irAE (31% vs. 10%) compared to anti PD-1 antibodies [22]. In particular, colitis (odds ratio (OR) 8.7) and hypophysitis (OR 6.5) were primarily observed with anti-CTLA-4 antibody preparations, pneumonitis (OR 6.4) and thyroiditis (OR 4.3) observed with anti-PD-1 antibody preparation [22]. In the CheckMate227 study, AEs were more prevalent in the nivolumab+ipilimumab group than in the nivolumab monotherapy group in groups with both all grades/grade-III and above (all grades 75.2% vs. 64.2%, grade-III and above 31.2% vs. 18.9%), and AEs for which treatment could not be continued were also reported in the combination group (12% vs. 6.9%) [23]. Other study has reported that the concomitant use of nivolumab+ipilimumab results in an earlier onset of irAE (particularly within 12 weeks) in comparison with nivolumab alone [24]. Thus, on using ICIs, more prudent measures for irAE are required than those used before.

### 4.3. Combination with Radiation Therapy

In 2019, ICIs with high efficacy were reported for the treatment of unresectable stage III NSCLC. As noted above, in a PACIFIC study (NCT02125461) testing the efficacy of CRT followed by continued durvalumab treatment as consolidation, durvalumab treatment drastically improved the PFS and OS in comparison with the control group (PFS: 16.8 mo vs. 5.6 mo, HR 0.52, The 24-month overall survival rate: 66.3% vs. 55.6%, HR 0.68), leading to a major development of locally advanced-stage standard-of-care in the first 20 y [25]. In terms of the frequency of pneumonitis concerns associated with concomitant use of radiation therapy (RT) and ICIs, although pneumonitis was more common in the durvalumab group in all grades (13.1% vs. 7.7%), only a slight difference was observed between the two groups in ≥ grade-III NSCLC (4.4% vs. 3.8%, respectively), resulting in no apparent increase in the risk of serious pneumonitis [18]. The PACIFIC trial was designed to use durvalumab as a consolidation therapy in the first 42 d after the completion of CRT, whereas the ongoing PACIFIC2 trial is testing the efficacy of combination therapy with CRT and durvalumab, rather than sequential therapy (NCT03519971). The JCOG1508 studies have compared platinum-based chemotherapy + RT + durvalumab vs. platinum-based chemotherapy + RT → surgical resection + durvalumab in unresectable stage III NSCLC with N2 nodal involvement and have tested the effectiveness of durvalumab in the combined modality therapy including surgery.

## 5. Effects on SCLC

SCLC is a smoking-associated cancer type accounting for 10%–15% of all lung cancers. The median overall survival is 15–20 mo for limited-stage disease and 8–13 mo for extensive-stage disease, and the 5-y survival rate is 20–25% for limited-stage disease and 2% for extensive-stage disease among patients. In a phase-2 study on advanced SCLC conducted in 2011, the effectiveness of combination therapy with ipilimumab and chemotherapy was explored; however, the primary endpoint, i.e., OS prolongation, was not achieved [26]. Although ICIs for SCLC have yielded less encouraging results; the results of an IMpower133 trial published in 2019 led to the approval of CBDCA+etoposide+atezolizumab for untreated extensive-stage SCLC, as described previously [27]. The KEYNOTE-604 trial also compared pembrolizumab+EP vs. placebo+EP for untreated extensive-stage SCLC; this trial reported a significant prolongation in the PFS but not OS. In the ongoing CASPIAN trial, a three-arm comparative trial of durvalumab+tremelimumab+EP or durvalumab+EP vs. EP for untreated extensive-stage SCLC, among 268 patients receiving combination therapy with durvalumab and standard chemotherapy and 269 patients receiving standard chemotherapy alone, the median OS was significantly prolonged from 10.3 mo in the standard chemotherapy group to 13.0 mo in the combination therapy group [28]. Thus, future studies are required to develop more combination therapies with ICIs for SCLC.

Moreover, SCLC, unlike NSCLC and malignant melanoma, is generally characterized by a lower rate of PD-L1 expression [29]; however, the association between PD-L1 incidence and ICI efficacy has not been determined in SCLC. Nonetheless, TMBs are reportedly associated with the efficacy of ICIs in CheckMate026 trial [5]. The CheckMate032 trial compared the efficacy of nivolumab monotherapy with that of combination therapy with nivolumab+ipilimumab for previously treated SCLC, and the overall OS was 5.7 mo vs. 4.7 mo with no significant difference in efficacy between the combination therapy and monotherapy [30]. However, subgroup analysis revealed that combination therapy with nivolumab+ipilimumab displayed a higher efficacy than nivolumab monotherapy [30]. However, few studies have investigated the therapeutic utility of PD-L1 and the TMB in SCLC, warranting further validation in future studies.

## 6. Biomarkers

Although the efficacy of ICIs has been confirmed, the response rate to single agents is not as high as that of molecular-targeting agents, and the establishment of biomarkers to predict effective responses to ICIs remains challenging.

As a biomarker for therapeutic efficacy, PD-L1 has been recently used in actual clinical practice. The KEYNOTE-001 (NCT01295827) trial reported that pembrolizumab was more effective in decreasing the incidence of PD-L1 by ≥ 50%, by 1% to 49%, and by < 1% [31]. Particularly in the ≥ 50% group, patients with very high PD-L1 levels (≥ 90%) presented an even higher response rate than those with 50%–89% expression levels and presented prolonged PFS (objective response rate (ORR) 60.0% vs. 32.7%, PFS 14.5 mo vs. 4.1 mo, HR 0.50) [32]. Other studies have reported that PD-L1 upregulation, regardless of monotherapy or combination therapy, is associated with an increased efficacy of pembrolizumab [16,17,33]. Furthermore, the efficacy of ICIs other than pembrolizumab are also associated with PD-L1 upregulation [19,34], and generally, PD-L1 upregulation is associated with a higher ICI efficacy. Based on these results, we recommend using pembrolizumab monotherapy as the first-line therapy for PD-L1 positive (≥ 1%) advanced-stage NSCLC. It is also recommended to use PD-1/PD-L1 inhibitors for advanced-stage NSCLC in immune-checkpoint inhibitor-naïve patients as the second-line therapy.

However, the incidence and effects do not necessarily coincide, suggesting that tumor cell heterogeneity is one of the causes along with the potential involvement of host immune evasion mechanisms not mediated by PD-1/PD-L1 [35]. PD-L1 is often debated to be an incomplete biomarker, and several studies have as attempted to develop new biomarkers and to combine PD-L1 with other biomarkers.

While ICIs exert antitumor effects by activating immune cells, tumor infiltrating lymphocytes (TILs) are important in mediating these effects, along with PD-L1 [36]. T cells included in TILs may be enriched with clones specific for tumor antigens, but are suppressed by an immunosuppressive tumor microenvironment, and so on, and thus, they are believed to be incapable of exerting effective anti-tumor responses [37]. Results from a KEYNOTE-061 trial (NCT02370498) examining the usefulness of pembrolizumab as a second-line treatment for advanced gastric cancer reported that the effect of pembrolizumab may be predicted by the combined positive score, the number of PD-L1 positive cells among tumor cells, lymphocytes, and macrophages divided by the total number of tumor cells multiplied by 100 [38]. When the tumor microenvironment is subtyped into four types according to the presence or absence of PD-L1 expression and the presence or absence of TILs, TIL-positive/PD-L1 positive Type I and TIL-positive/PD-L1 negative Type IV are considered as “Hot tumors” in which anti-PD-1 antibodies are effective alone or in combination [36]. The usefulness of PD-L1 as a TIL biomarker has been reported in breast cancer [39], and similar studies are expected in future.

Other potential biomarkers include the total number of genetic mutations in cells, called tumor mutation burden (TMB). Mutated genes invariably yield mutated proteins, which are recognized as non-self by immune cells; hence, cells containing numerous mutated proteins are more susceptible to be attacked by immune cells, and cells with a high TMB are considered to display a more effective response to ICIs. In general, the TMB tends to be higher among smokers [40], and the relatively higher efficacy of ICI among smokers is speculated to result from the TMB [12,41]. In the 2017 CheckMate026 trial (NCT02041533), nivolumab was compared with platinum-based chemotherapy for first-line treatment of advanced-stage NSCLC with PD-L1 ≥ 5%, and nivolumab did not demonstrate superiority for the primary endpoint, PFS [5]. However, this study on exploratory TMB stratification analysis suggested that ICIs may be more effective in the high TMB group (TMB ≥ 243 nonsynonymous mutations) than in the low TMB group (TMB < 243 nonsynonymous mutations) (9.7 mo vs. 5.8 mo HR 0.62). A CheckMate227 trial (NCT02477826) (2019) comparing the efficacy of nivolumab+ipilimumab combination therapy vs. nivolumab monotherapy vs. platinum-based chemotherapy with TMB as a biomarker demonstrated the superiority of nivolumab+ipilimumab combination therapy to that of platinum-based chemotherapy; however, this tendency was stronger in the group with a high TMB (≥10 Mut/Mb) (global high: 23.0 mo vs. 16.4 mo, HR 0.68; low: 16.2 mo vs. 12.6 mo, HR 0.7, PD-L1 < 1% high: 20.4 mo vs. 11.2 mo, HR 0.51, low: 15.5 mo vs. 13.0 mo, HR 0.69) [23]. A CheckMate568 trial (NCT02659059) evaluated the safety and efficacy of concomitant nivolumab+ipilimumab combination therapy in the same year, reporting that the high-TMB group had a significantly prolonged PFS (7.1 mo vs. 2.6 mo) [42]. Again, the results showed that TMBs and ORRs were predominantly associated in the group with PD-L1 < 1% (AUC 0.90) [42]. Consistent with the results of CheckMate568, the ORR with nivolumab plus ipilimumab in CheckMate227 was higher for tumors with PD-L1 > 1% compared with that for tumors with PD-L1 < 1% [21,42]. However, the relationship between the PD-L1 biomarker and efficacy of the combination of nivolumab and low-dose ipilimumab is complex, as in CheckMate227, there was a similar survival advantage for nivolumab and low-dose ipilimumab compared with that for standard chemotherapy in PD-L1-positive and PD-L1-negative tumors. Thus, the TMB (particularly in cases with PD-L1 < 1%) is reportedly associated with the efficacy of ICIs. At present, clinical trials considering TMBs as biomarkers are underway for atezolizumab (BFAST study: NCT03178552), and future studies are expected to yield clinically significant results.

The other reported potential benefits of these biomarkers include the prognostic nutritional index (PNI) and its association with the frequency of irAEs, neutrophil-to-lymphocyte ratio (NLR) [43], enterobacterial status [44,45], and early reduction in tumor markers [46].

Although the PNI was proposed as a predictor of surgical risk in the 1980s, it has been subsequently considered a useful marker to predict the efficacy of drugs to treat malignant diseases. Studies have reported that ICIs are more effective among patients with a high PNI, i.e., high nutritional status [47]. Further, numerous studies have reported that ICIs are more effective among patients with irAE [22,48], and that the management of adverse events is potentially important for the continuation of effective treatment.

## 7. Long-Term Survival

A major difference between ICIs and previously reported anticancer drugs is a substantial increase in long-term survival. On pooled analysis of the CheckMate017 and CheckMate057 trials (2019), both of which compared the efficacy of DTX and nivolumab as second-line therapy for NSCLC, the 5-y survival rate of nivolumab was 13.4%; DTX, 2.6% [49]. A 5-y survival rate of > 10% has not yet been achieved using previously reported cytocidal anticancer drugs, and ICIs resulted in a more prolonged long-term survival than conventional anticancer drugs. Furthermore, at 5 y, nivolumab treatment resulted in a response among 32.2% of patients, while no patients responded to DTX.

A follow-up report from the KEYNOTE-001 trial, a phase-I study on pembrolizumab, also reported a 5-y survival rate of 29.6% in the untreated, high PD-L1 group [50]. Notwithstanding a high long-term survival rate, the expression of PD-L1 and the frequency of TMBs are considered suitable predictors. Rizvi et al. reported that 62 patients with NSCLC, who received an ICI and acquired a PFS of ≥18 mo, presented significantly better outcomes on the basis of both PD-L1 and TMB in comparison with untreated patients (rate of PD-L1 TPS ≥ 50%: 43% vs. 23% TMB: 12.24 vs. 6.34 Mut/Mb) [51].

Though not observed in a majority of patients, treatment with immune checkpoint inhibitors may result in long-term survival, and establishment of biomarkers on long-term surviving cases is desirable.

## 8. Challenges Associated with ICIs

The development of ICIs is not without its challenges. Other than the aforementioned biomarkers, the following challenges may be considered.

### 8.1. Treatment of Patients Harboring a Driver Mutation

ICIs are clearly less effective among patients harboring driver mutations. Among other EGFR mutations, exon del19 is reported to result in a lower PFS than L858R on ICI treatment (del19 HR 0.449 *p* < 0.001, L858R HR 0.578 *p* = 0.001) [52]. Among these, the PFS, among patients harboring these mutations, was significantly higher when the PD-L1 expression rate was compared between the negative group (0%) and the positive group (≥1%) (2.8 mo vs. 1.7 mo) results [52], suggesting that PD-L1 expression rate may be related to the efficacy of ICIs, even among patients harboring driver mutations. Regimens combining atezolizumab with CBDCA+PTX+BEV in the IMpower150 trial were also effective among patients harboring driver mutations upon subgroup analysis [19], and we believe that they hold promise as second-line treatment candidates upon using molecular-targeted agents. WJCOG8515L trial (UMIN000021133) have compared nivolumab with CBDCA+PEM in EGFR-TKI post-treatment NSCLC resistant cases through mechanisms other than T790M, and we believe that use of ICI for patients harboring driver mutations would be a future challenge.

### 8.2. Applicability Among Patients with a History of Interstitial Pneumonia or Autoimmune Disease

Managing irAE is of great importance with the use of ICIs. Characteristic adverse events that are less common but not experienced with cytotoxic anticancer drugs or molecular-targeted agents have become evident. Regarding disease management after manifestation, close cooperation among medical care departments is important as the AEs seem to be caused by immune activity in all organs.

Regarding the risk factors for irAE, ICIs activate the autoimmune system and induce antitumor effects. In patients with a history of autoimmune disease or interstitial pneumonitis, exacerbation of these underlying diseases or an increased incidence of irAE are worrisome and thus, cautious administration is recommended.

Furthermore, a higher incidence of smoking, and numerous cases with complications of smoking-related interstitial pneumonia have been reported. The use of ICIs among patients with interstitial pneumonia or autoimmune diseases is often excluded in clinical trials, and a few retrospective data have been reported.

Fujimoto et al. reported that 2 of 18 patients with mild-to-moderate idiopathic interstitial pneumonia had grade-II pneumonitis and that pneumonitis was alleviated in 6 patients with moderate pneumonitis upon nivolumab treatment [53]. The incidence of pneumonitis with previous ICIs did not significantly increase as the all-grade incidence of pneumonitis in ICIs ranged from 5% to 10%. On the contrary, Kanai et al. reported that the incidence of pneumonitis upon nivolumab treatment was significantly higher in the group with a history of interstitial pneumonia (31% vs. 12%), and 62% vs. 45% for grade-III or higher was associated with higher risks in the group with a history of interstitial pneumonia [54]. No deaths due to pneumonitis were recorded in these reports.

Leonardi et al. reported that treatment with ICIs alone among patients with autoimmune diseases resulted in disease exacerbation in 23% of patients, of which 32% required treatment with steroids [55]. Moreover, 38% developed some form of irAE, of which 26% were grade-III or higher [55]. Overall, 55% of patients experienced exacerbations of irAE, autoimmune disease, or both, and the incidence of irAE was similar to that in patients without autoimmune disease [55].

These reports on patients with a history of interstitial pneumonia or autoimmune disease provide retrospective data; however, it is considered necessary to exclude patients who judge the use of ICIs to be inappropriate based on their condition.

Studies wherein patients with interstitial pneumonia or autoimmune disease were administered ICIs have not provided adequate data on their safety and efficacy, and caution should be exercised with their use. In particular, the benefit for patients with high PD-L1 expression levels seems to be non-negligible, and individualized correspondence is required considering the balance with risk.

### 8.3. Co-Administration of Steroids

Tumor-bearing patients often receive steroids as symptomatic treatment for worsening systemic symptoms and symptoms due to cancer progression. In general, steroids are routinely administered as antiemetics during platinum-based chemotherapy. However, steroids may reduce the effects of ICIs by suppressing immune responses induced by IL-2 and CD8-positive T cells [56,57], and increasing Tregs [58,59].

Ricciuti et al. reported that patients receiving PSL-equivalent steroids at ≥10 mg on the initiation of ICI therapy had a significantly shorter survival (PFS 2.0 mo vs. 3.4 mo, HR 1.3; OS 4.9 mo vs. 11.2 mo, HR 1.7) than those receiving ICI-equivalent steroids at ≤10 mg [60]. On the contrary, the use of steroids for therapeutic purposes to counter irAE occurring during ICI treatment does not impair the efficacy of ICI [61,62]. Thus, co-administration of steroids during ICI therapy remains a future challenge for lung cancer treatment.

## 9. Conclusions

ICIs have transformed the treatment of lung cancer. Although the number of patients with long-term survival after ICI treatment is significantly greater than that with previous therapies, such cases are limited, and novel therapies such as methods of selection and combination therapies that enhance efficacy remain an important issue to be resolved. The development of predictive factors for immunotherapy is crucial with regard to the efficacy of future treatment gains. Although both PD-L1 and TMBs may be helpful in case selection, it is now clear that resistance can develop by more than one mechanism. In future, further optimized treatments can be expected by combining cancer genomic information with the assessment results of cellular components from the tumor microenvironment. Although immunochemotherapy has shown great success in the treatment of lung cancer, it is expected that treatment will be individualized further on a case-by-case basis in future and will be improved by the development of combination treatments with targeted or cellular therapies, or new combinations of immunotherapies. Future challenges will likely involve targeting the correct immunotherapy to the correct immune microenvironment at an appropriate time. On the contrary, although not detailed in this article, the side effects of immune checkpoint inhibitors are very different from those of conventional cytocidal anticancer drugs and molecularly targeted drugs, spanning various organs including the skin and the digestive, respiratory, thyroid, and pituitary glands. These are considered side effects due to excessive autoimmune reactions, which are relatively infrequent and if present, are usually mild, allowing continued treatment with immune checkpoint inhibitors under careful management. However, adverse event management during treatment requires caution, as moderate to high immune-related adverse events are associated with markedly reduced organ function and quality of life, and fatal consequences have also been reported. Establishment of more appropriate usage methods such as the development of biomarkers and of combined immunotherapy is highly desired in the future.

## Figures and Tables

**Table 1:** Trials of ICIs for advanced stage NSCLC.

Trial	Patient Population	Treatment Regimen	Primary Outcome Results
CheckMate 017	Stage IIIB/IV squamous NSCLC; disease recurrence after platinum-based chemotherapy	Nivolumab	Median OS: 9.2 months (95% CI: 7.3–13.3); 12 months OS: 42% (95% CI: 34–50%)
CheckMate 057	Stage IIIB/IV non-squamous NSCLC; disease recurrence after platinum-based chemotherapy	Nivolumab	Median OS: 12.2 months (95% CI: 9.7–15.1); 18 months OS: 39% (95% CI: 34–45%)
OAK	Stage IIIB/IV; disease progression after platinum-based chemotherapy	Atezolizumab	Median OS: 13.8 months (95% CI: 11.8–15.7); PP-ITT; improved OS/PFS in patients with PD-L1 expression > 1%
IMpower 150	Stage IIIB/IV; untreated metastatic non-squamous NSCLC	Chemotherapy + Bevacizumab ± Atezolizumab	Median PFS: 8.3 months (95% CI: 7.7–9.8); Median OS: 19.8 months (95% CI: 17.4–24.2)
KEYNOTE 024	Stage IV; untreated disease; PD-L1 expression > 50%	Pembrolizumab	Median PFS: 10.3 months (95% CI: 6.7–NR); 6 months PFS: 62.1% (95% CI: 53.8–69.4%)
KEYNOTE 189	Stage IIIB/IV; untreated metastatic non-squamous NSCLC	Chemotherapy ± Pembrolizumab	Median OS: 22.0 months (95% CI: 19.5–25.2); 12 months OS: 69.2% (95% CI: 64.1–73.8%)
KEYNOTE 407	Stage IIIB/IV; untreated metastatic squamous NSCLC	Chemotherapy ± Pembrolizumab	Median OS: 15.9 months (95% CI: 13.2–NR); 12 months OS: 65.2% (95% CI: 57.7–71.6%)

ICIs: Immune checkpoint inhibitors; NSCLC: non-small cell lung cancer; PD-L1: programmed cell-death ligand 1; OS: overall survival; PFS: progression-free survival; NR: not reached.

## References

[1] Bray F., Ferlay J., Soerjomataram I., Siegel R.L., Torre L.A., Jemal A. (2018). Global cancer statistics 2018: GLOBOCAN estimates of incidence and mortality worldwide for 36 cancers in 185 countries. CA A Cancer J. Clin..

[2] Chen D., Mellman I. (2013). Oncology Meets Immunology: The Cancer-Immunity Cycle. Immunity.

[3] Schreiber R.D., Old L.J., Smyth M.J. (2011). Cancer Immunoediting: Integrating Immunity’s Roles in Cancer Suppression and Promotion. Science.

[4] Motzer R.J., Tannir N.M., McDermott D.F., Frontera O.A., Melichar B., Choueiri T.K., Plimack E.R., Barthelemy P., Porta C., George S. (2018). Nivolumab plus Ipilimumab versus Sunitinib in Advanced Renal-Cell Carcinoma. N. Engl. J. Med..

[5] Carbone D.P., Reck M., Paz-Ares L., Creelan B., Horn L., Steins M., Felip E., Heuvel M.M.V.D., Ciuleanu T.-E., Badin F. (2017). First-Line Nivolumab in Stage IV or Recurrent Non–Small-Cell Lung Cancer. N. Engl. J. Med..

[6] Hui E., Cheung J., Zhu J., Su X., Taylor M.J., Wallweber H.A., Sasmal D.K., Huang J., Kim J.M., Mellman I. (2017). T cell costimulatory receptor CD28 is a primary target for PD-1–mediated inhibition. Science.

[7] Sansom D. (2000). CD28, CTLA-4 and their ligands: Who does what and to whom?. Immunology.

[8] Rowshanravan B., Halliday N., Sansom D. (2018). CTLA-4: A moving target in immunotherapy. Blood.

[9] Malas S., Harrasser M., Lacy K.E., Karagiannis S.N. (2014). Antibody therapies for melanoma: New and emerging opportunities to activate immunity (Review). Oncol. Rep..

[10] Walunas T.L., Lenschow D.J., Bakker C.Y., Linsley P.S., Freeman G.J., Green J.M., Thompson C.B., Bluestone J.A. (1994). CTLA-4 can function as a negative regulator of T cell activation. Immunity.

[11] Brahmer J., Reckamp K.L., Baas P., Crino L., Eberhardt W.E., Poddubskaya E.V., Antonia S., Pluzanski A., Vokes E.E., Holgado E. (2015). Nivolumab versus Docetaxel in Advanced Squamous-Cell Non-Small-Cell Lung Cancer. N. Engl. J. Med..

[12] Borghaei H., Paz-Ares L., Horn L.A., Spigel D.R., Steins M., Ready N.E., Chow L.Q., Vokes E.E., Felip E., Holgado E. (2015). Nivolumab versus Docetaxel in Advanced Nonsquamous Non-Small-Cell Lung Cancer. N. Engl. J. Med..

[13] Herbst R.S., Baas P., Kim N.-W., Felip E., Pérez-Gracia J.L., Han J.-Y., Molina J., Kim J.-H., Arvis C.D., Ahn M.-J. (2016). Pembrolizumab versus docetaxel for previously treated, PD-L1-positive, advanced non-small-cell lung cancer (KEYNOTE-010): A randomised controlled trial. Lancet.

[14] Rittmeyer A., Barlesi F., Waterkamp D., Park K., Ciardiello F., Von Pawel J., Gadgeel S.M., Hida T., Kowalski D., Dols M.C. (2017). Atezolizumab versus docetaxel in patients with previously treated non-small-cell lung cancer (OAK): A phase 3, open-label, multicentre randomised controlled trial. Lancet.

[15] Reck M., Rodríguez-Abreu D., Robinson A.G., Hui R., Csőszi T., Fülöp A., Gottfried M., Peled N., Tafreshi A., Cuffe S. (2016). Pembrolizumab versus Chemotherapy for PD-L1–Positive Non–Small-Cell Lung Cancer. N. Engl. J. Med..

[16] Gandhi L., Rodríguez-Abreu D., Gadgeel S., Esteban E., Felip E., De Angelis F., Dómine M., Clingan P., Hochmair M.J., Powell S.F. (2018). Pembrolizumab plus Chemotherapy in Metastatic Non–Small-Cell Lung Cancer. N. Engl. J. Med..

[17] Paz-Ares L., Luft A., Vicente D., Tafreshi A., Gümüş M., Mazieres J., Hermes B., Çay Şenler F., Csőszi T., Fülöp A. (2018). Pembrolizumab plus Chemotherapy for Squamous Non–Small-Cell Lung Cancer. N. Engl. J. Med..

[18] Antonia S.J., Villegas A., Daniel D., Vicente D., Murakami S., Hui R., Yokoi T., Chiappori A., Lee K.H., De Wit M. (2017). Durvalumab after Chemoradiotherapy in Stage III Non-Small-Cell Lung Cancer. N. Engl. J. Med..

[19] Socinski M.A., Jotte R.M., Cappuzzo F., Orlandi F., Stroyakovskiy D., Nogami N., Rodríguez-Abreu D., Moro-Sibilot D., Thomas C.A., Barlesi F. (2018). Atezolizumab for First-Line Treatment of Metastatic Nonsquamous NSCLC. N. Engl. J. Med..

[20] West H., McCleod M., Hussein M., Morabito A., Rittmeyer A., Conter H.J., Kopp H.-G., Daniel D., McCune S., Mekhail T. (2019). Atezolizumab in combination with carboplatin plus nab-paclitaxel chemotherapy compared with chemotherapy alone as first-line treatment for metastatic non-squamous non-small-cell lung cancer (IMpower130): A multicentre, randomised, open-label, phase 3 trial. Lancet Oncol..

[21] Hellmann M.D., Paz-Ares L., Caro R.B., Zurawski B., Kim S.-W., Costa E.C., Park K., Alexandru A., Lupinacci L., Jimenez E.D.L.M. (2019). Nivolumab plus Ipilimumab in Advanced Non–Small-Cell Lung Cancer. N. Engl. J. Med..

[22] Khoja L., Day D., Chen T.W.-W., Siu L.L., Hansen A. (2017). Tumour- and class-specific patterns of immune-related adverse events of immune checkpoint inhibitors: A systematic review. Ann. Oncol..

[23] Hellmann M.D., Ciuleanu T.-E., Pluzanski A., Lee J.S., Otterson G.A., Audigier-Valette C., Minenza E., Linardou H., Burgers S., Salman P. (2018). Nivolumab plus Ipilimumab in Lung Cancer with a High Tumor Mutational Burden. N. Engl. J. Med..

[24] Haanen J.B.A.G., Carbonnel F., Robert C., Kerr K.M., Peters S., Larkin J., Jordan K., ESMO Guidelines Committee (2017). Management of toxicities from immunotherapy: ESMO Clinical Practice Guidelines for diagnosis, treatment and follow-up. Ann. Oncol..

[25] Antonia S.J., Villegas A., Daniel D., Vicente D., Murakami S., Hui R., Kurata T., Chiappori A., Lee K.H., De Wit M. (2018). Overall Survival with Durvalumab after Chemoradiotherapy in Stage III NSCLC. N. Engl. J. Med..

[26] Reck M., Luft A., Szczesna A., Havel L., Kim S.-W., Akerley W., Pietanza M.C., Wu Y.-L., Zielinski C., Thomas M. (2016). Phase III Randomized Trial of Ipilimumab Plus Etoposide and Platinum Versus Placebo Plus Etoposide and Platinum in Extensive-Stage Small-Cell Lung Cancer. J. Clin. Oncol..

[27] Horn L., Mansfield A., Szczęsna A., Havel L., Krzakowski M., Hochmair M.J., Huemer F., Losonczy G., Johnson M.L., Nishio M. (2018). First-Line Atezolizumab plus Chemotherapy in Extensive-Stage Small-Cell Lung Cancer. N. Engl. J. Med..

[28] Paz-Ares L., Dvorkin M., Chen Y., Reinmuth N., Hotta K., Trukhin D., Statsenko G., Hochmair M.J., Özgüroğlu M., Ji J.H. (2019). Durvalumab plus platinum–etoposide versus platinum–etoposide in first-line treatment of extensive-stage small-cell lung cancer (CASPIAN): A randomised, controlled, open-label, phase 3 trial. Lancet.

[29] Yoshimura A., Yamada T., Miyagawa-Hayashino A., Sonobe Y., Imabayashi T., Yamada T., Okada S., Shimamoto T., Chihara Y., Iwasaku M. (2019). Comparing three different anti-PD-L1 antibodies for immunohistochemical evaluation of small cell lung cancer. Lung Cancer.

[30] Ready N., Ott P.A., Hellmann M.D., Zugazagoitia J., Hann C.L., De Braud F., Antonia S.J., Ascierto P.A., Moreno V., Atmaca A. (2020). Nivolumab Monotherapy and Nivolumab Plus Ipilimumab in Recurrent Small Cell Lung Cancer: Results From the CheckMate 032 Randomized Cohort. J. Thorac. Oncol..

[31] Hui R., Garon E.B., Goldman J.W., Leighl N.B., Hellmann M.D., Patnaik A., Gandhi L., Eder J.P., Ahn M.-J., Horn L. (2017). Pembrolizumab as first-line therapy for patients with PD-L1-positive advanced non-small cell lung cancer: A phase 1 trial. Ann. Oncol..

[32] Aguilar E., Ricciuti B., Gainor J., Kehl K., Kravets S., Dahlberg S., Nishino M., Sholl L., Adeni A., Subegdjo S. (2019). Outcomes to first-line pembrolizumab in patients with non-small-cell lung cancer and very high PD-L1 expression. Ann. Oncol..

[33] Mok T.S., Wu Y.-L., Kudaba I., Kowalski D.M., Cho B.C., Turna H.Z., Castro G., Srimuninnimit V., Laktionov K.P., Bondarenko I. (2019). Pembrolizumab versus chemotherapy for previously untreated, PD-L1-expressing, locally advanced or metastatic non-small-cell lung cancer (KEYNOTE-042): A randomised, open-label, controlled, phase 3 trial. Lancet.

[34] Park S., Choi Y.-D., Kim J., Kho B.-G., Park C.-K., Oh I.-J., Kim Y.-C. (2019). Efficacy of immune checkpoint inhibitors according to PD-L1 tumor proportion scores in non-small cell lung cancer. Thorac. Cancer.

[35] McGranahan N., Furness A.J.S., Rosenthal R., Ramskov S., Lyngaa R., Saini S.K., Jamal-Hanjani M., Wilson G.A., Birkbak N.J., Hiley C. (2016). Clonal neoantigens elicit T cell immunoreactivity and sensitivity to immune checkpoint blockade. Science.

[36] Teng M.W., Ngiow S.F., Ribas A., Smyth M.J. (2015). Classifying Cancers Based on T-cell Infiltration and PD-L1. Cancer Res..

[37] Rosenberg S.A., Restifo N.P. (2015). Adoptive cell transfer as personalized immunotherapy for human cancer. Science.

[38] Shitara K., De Braud F., Mandalà M., Fornaro L., Olesiński T., Caglevic C., Muro K., Mansoor W., McDermott R., Chen X. (2018). Pembrolizumab versus paclitaxel for previously treated, advanced gastric or gastro-oesophageal junction cancer (KEYNOTE-061): A randomised, open-label, controlled, phase 3 trial. Lancet.

[39] Adams S., Schmid P., Rugo H., Winer E., Loirat D., Awada A., Cescon D., Iwata H., Campone M., Nanda R. (2019). Pembrolizumab monotherapy for previously treated metastatic triple-negative breast cancer: Cohort A of the phase II KEYNOTE-086 study. Ann. Oncol..

[40] Gibbons D.L., Byers L.A., Kurie J.M. (2014). Smoking, p53 mutation, and lung cancer. Mol. Cancer Res..

[41] Gainor J.F., Shaw A.T., Sequist L.V., Fu X., Azzoli C.G., Piotrowska Z., Huynh T.G., Zhao L., Fulton L., Schultz K.R. (2016). EGFR Mutations and ALK Rearrangements Are Associated with Low Response Rates to PD-1 Pathway Blockade in Non-Small Cell Lung Cancer: A Retrospective Analysis. Clin. Cancer Res..

[42] Ready N., Hellmann M.D., Awad M.M., Otterson G.A., Gutierrez M., Gainor J.F., Borghaei H., Jolivet J., Horn L., Mates M. (2019). First-Line Nivolumab Plus Ipilimumab in Advanced Non–Small-Cell Lung Cancer (CheckMate 568): Outcomes by Programmed Death Ligand 1 and Tumor Mutational Burden as Biomarkers. J. Clin. Oncol..

[43] Jeyakumar G., Kim S., Bumma N., Landry C., Silski C., Suisham S., Dickow B., Heath E.I., Fontana J., Vaishampayan U. (2017). Neutrophil lymphocyte ratio and duration of prior anti-angiogenic therapy as biomarkers in metastatic RCC receiving immune checkpoint inhibitor therapy. J. Immunother. Cancer.

[44] Gopalakrishnan V., Spencer C.N., Nezi L., Reuben A., Andrews M.C., Karpinets T.V., Prieto P.A., Vicente D., Hoffman K., Wei S.C. (2017). Gut microbiome modulates response to anti–PD-1 immunotherapy in melanoma patients. Science.

[45] Sears C., Pardoll E.M. (2018). The intestinal microbiome influences checkpoint blockade. Nat. Med..

[46] Lang D., Horner A., Brehm E., Akbari K., Hergan B., Langer K., Asel C., Scala M., Kaiser B., Lamprecht B. (2019). Early serum tumor marker dynamics predict progression-free and overall survival in single PD-1/PD-L1 inhibitor treated advanced NSCLC—A retrospective cohort study. Lung Cancer.

[47] Shoji F., Takeoka H., Kozuma Y., Toyokawa G., Yamazaki K., Ichiki M., Takeo S. (2019). Pretreatment prognostic nutritional index as a novel biomarker in non-small cell lung cancer patients treated with immune checkpoint inhibitors. Lung Cancer.

[48] Maher V.E., Fernandes L.L., Weinstock C., Tang S., Agarwal S., Brave M., Ning Y.-M., Singh H., Suzman D., Xu J. (2019). Analysis of the Association Between Adverse Events and Outcome in Patients Receiving a Programmed Death Protein 1 or Programmed Death Ligand 1 Antibody. J. Clin. Oncol..

[49] Gettinger S., Borghaei H., Brahmer J., Chow L., Burgio M., Carpeno J.D.C., Pluzanski A., Arrieta O., Frontera O.A., Chiari R. (2019). OA14.04 Five-Year Outcomes From the Randomized, Phase 3 Trials CheckMate 017/057: Nivolumab vs. Docetaxel in Previously Treated NSCLC. J. Thorac. Oncol..

[50] Garon E.B., Hellmann M.D., Rizvi N.A., Carcereny E., Leighl N.B., Ahn M.-J., Eder J.P., Balmanoukian A.S., Aggarwal C., Horn L. (2019). Five-Year Overall Survival for Patients With Advanced Non‒Small-Cell Lung Cancer Treated With Pembrolizumab: Results From the Phase I KEYNOTE-001 Study. J. Clin. Oncol..

[51] Rizvi H., Plodkowski A.J., Tenet M., Halpenny D., Long N., Sauter J.L., Sanchez-Vega F., Chatila W., Schultz N., Ladanyi M. (2018). Clinical and molecular features predicting long-term response (LTR) to anti-PD-(L)1 based therapy in patients with NSCLC. J. Clin. Oncol..

[52] Hastings K., Yu H., Wei W., Sanchez-Vega F., Deveaux M., Choi J., Rizvi H., Lisberg A., Truini A., Lydon C. (2019). EGFR mutation subtypes and response to immune checkpoint blockade treatment in non-small-cell lung cancer. Ann. Oncol..

[53] Fujimoto D., Yomota M., Sekine A., Morita M., Morimoto T., Hosomi Y., Ogura T., Tomioka H., Tomii K. (2019). Nivolumab for advanced non-small cell lung cancer patients with mild idiopathic interstitial pneumonia: A multicenter, open-label single-arm phase II trial. Lung Cancer.

[54] Kanai O., Kim Y.H., Demura Y., Kanai M., Ito T., Fujita K., Yoshida H., Akai M., Mio T., Hirai T. (2018). Efficacy and safety of nivolumab in non-small cell lung cancer with preexisting interstitial lung disease. Thorac. Cancer.

[55] Leonardi G.C., Gainor J.F., Altan M., Kravets S., Dahlberg S.E., Gedmintas L., Azimi R., Rizvi H., Riess J.W., Hellmann M.D. (2018). Safety of Programmed Death–1 Pathway Inhibitors Among Patients With Non–Small-Cell Lung Cancer and Preexisting Autoimmune Disorders. J. Clin. Oncol..

[56] Bianchi M., Meng C., Ivashkiv L.B. (2000). Inhibition of IL-2-induced Jak-STAT signaling by glucocorticoids. Proc. Natl. Acad. Sci. USA.

[57] Im S.J., Hashimoto M., Gerner M.Y., Lee J., Kissick H.T., Burger M.C., Shan Q., Hale J.S., Lee J., Nasti T.H. (2016). Defining CD8+ T cells that provide the proliferative burst after PD-1 therapy. Nature.

[58] Chen X., Murakami T., Oppenheim J.J., Howard O. (2004). Differential response of murine CD4+CD25+and CD4+CD25-T cells to dexamethasone-induced cell death. Eur. J. Immunol..

[59] Chen X., Oppenheim J.J., Ortaldo J.R., Howard O., Winkler-Pickett R.T. (2006). Glucocorticoid amplifies IL-2-dependent expansion of functional FoxP3+CD4+CD25+ T regulatory cellsin vivo and enhances their capacity to suppress EAE. Eur. J. Immunol..

[60] Ricciuti B., Dahlberg S.E., Adeni A., Sholl L.M., Nishino M., Awad M.M. (2019). Immune Checkpoint Inhibitor Outcomes for Patients with Non–Small-Cell Lung Cancer Receiving Baseline Corticosteroids for Palliative Versus Nonpalliative Indications. J. Clin. Oncol..

[61] Santini F.C., Rizvi H., Plodkowski A.J., Ni A., Lacouture M.E., Gambarin-Gelwan M., Wilkins O., Panora E., Halpenny D.F., Long N.M. (2018). Safety and Efficacy of Re-treating with Immunotherapy after Immune-Related Adverse Events in Patients with NSCLC. Cancer Immunol. Res..

[62] Horvat T., Adel N.G., Dang T.-O., Momtaz P., Postow M.A., Callahan M.K., Carvajal R.D., Dickson M.A., D’Angelo S.P., Woo K.M. (2015). Immune-Related Adverse Events, Need for Systemic Immunosuppression, and Effects on Survival and Time to Treatment Failure in Patients With Melanoma Treated With Ipilimumab at Memorial Sloan Kettering Cancer Center. J. Clin. Oncol..

